# Uptake of evidence by physicians: De-adoption of erythropoiesis-stimulating agents after the TREAT trial

**DOI:** 10.1186/s12882-021-02491-y

**Published:** 2021-08-21

**Authors:** Khoa Vu, Jiani Zhou, Alexander Everhart, Nihar Desai, Jeph Herrin, Anupam B. Jena, Joseph S. Ross, Nilay D. Shah, Pinar Karaca-Mandic

**Affiliations:** 1 Department of Applied Economics, University of Minnesota, 1994 Buford Avenue, St. Paul, MN 55108 USA; 2 University of Minnesota School of Public Health, 420 Delaware St. SE, Minneapolis, MN 55455 USA; 3 Cardiovascular Medicine, Yale School of Medicine, 15 York Street, PO Box 208017, New Haven, CT 06520-8017 USA; 4 Yale School of Medicine, PO Box 2254, Charlottesville, Virginia 22902 USA; 5 Harvard Medical School and National Bureau of Economic Research, 180 Longwood Avenue, Boston, MA 02115 USA; 6 Yale School of Medicine, General Internal Medicine, P.O. Box 208093, New Haven, CT 06520-8093 USA; 7 Mayo Clinic, Rochester, MN 55905 USA; 8 National Bureau of Economic Research and OptumLabs Visiting Fellow, University of Minnesota Carlson School of Management, 321 19th Avenue South, Minneapolis, MN 55455 USA

**Keywords:** De-adoption, Physician prescribing, Medication utilization, Medical safety

## Abstract

**Background:**

Variation in de-adoption of ineffective or unsafe treatments is not well-understood. We examined de-adoption of erythropoiesis-stimulating agents (ESA) in anemia treatment among patients with chronic kidney disease (CKD) following new clinical evidence of harm and ineffectiveness (the TREAT trial) and the FDA’s revision of its safety warning.

**Method:**

We used a segmented regression approach to estimate changes in use of epoetin alfa (EPO) and darbepoetin alfa (DPO) in the commercial, Medicare Advantage (MA) and Medicare fee-for-service (FFS) populations. We also examined how changes in both trends and levels of use were associated with physicians’ characteristics.

**Results:**

Use of DPO and EPO declined over the study period. There were no consistent changes in DPO trend across insurance groups, but the level of DPO use decreased right after the FDA revision in all groups. The decline in EPO use trend was faster after the TREAT trial for all groups. Nephrologists were largely more responsive to evidence than primary care physicians. Differences by physician’s gender, and age were not consistent across insurance populations and types of ESA.

**Conclusions:**

Physician specialty has a dominant role in prescribing decision, and that specializations with higher use of treatment (nephrologists) were more responsive to new evidence of unsafety and ineffectiveness.

**Supplementary Information:**

The online version contains supplementary material available at 10.1186/s12882-021-02491-y.

## Background

Anemia is common among patients with chronic kidney disease (CKD), and erythropoiesis-stimulating agents (ESA) are commonly used to stimulate bone marrow to produce red blood cells, improving anemia symptoms and preventing the need for blood transfusion [1]. The treatment is typically initiated when a patient’s level of hemoglobin concentration is too low, and the treatment is used until the hemoglobin concentration reaches a “safe” range [2]. The main types of ESA in the U.S. are epoetin alfa (EPO) [1] and darbepoetin alfa (DPO) [3], which mainly differ in how frequently the drug is administered to patients [2]. DPO is a newer, synthetic form of naturally-occurring erythropoietin that has a longer duration of action, requiring less frequent administration [4]. EPO is usually administered three times a week while DPO is administered once a week up to once every a month [5, 6].

The use of ESA therapy for anemia in CKD patients is common in many countries – Wong et al. [7] estimated that 48% patients with a hemoglobin level < 10 g/dL in the US, 58% in Brazil, 66% in France, and 70% in Germany were prescribed an ESA or iron in the 3 months following Hb measurement. Clinical evidence over the past decades, however, has suggested that using ESA to target high hemoglobin levels (i.e., ≥ 13 g/dL) might be unsafe. The first study to raise safety concerns was the Normal Hematocrit Cardiac Trial (NHCT) [8] in hemodialysis patients in 1998 (targeting 14 versus 10 g/dL), followed by two other studies in patients with non-dialysis CKD in 2006 – the Correction of Hemoglobin and Outcomes in Renal Insuggeiciency [9] (CHOIR; targeting 13.5 versus 11.3 g/dL) and Cardiovascular Risk Reduction by Early Anemia Treatment with Epoetin beta [10] (CREATE; targeting 13.5 g/dL versus 11.5 g/dL). In October 2009, the Trial to Reduce Cardiovascular Events with Aranesp Therapy (TREAT), a large placebo-controlled study, found that DPO treatment targeting a hemoglobin level of 13 g/dL did not reduce the risk of either death, cardiovascular, or renal events, but resulted in a higher risk of stroke and less frequent cardiac revascularization, compared to using DPO when hemoglobin level fell below 9 g/dL [11]. In response, the U.S. Food and Drug Administration (FDA) revised its original black-box warnings in June 2011, recommending using the lowest dose of ESA necessary to reduce the need for blood transfusion, and reducing dose or interrupting the treatment when Hb level exceeds 10 g/dL [12]. Other health authorities and organizations in the U.S. and other countries have also adjusted practice guidelines to reflect this risk or changed the payment rules regarding ESA treatment [13–17].

Several prior studies have found substantial de-adoption of ESA treatment in the US following the CREATE and CHOIR publications [18, 19], the TREAT trial [20, 21], the FDA revision [21], and the change of payment rules [22, 23]. Other studies have also documented decreases of ESA treatment during the same period in other countries such as Canada, Germany, and Japan [17, 24]. These studies have not examined physician patterns of de-adopting ESA treatment, nor whether these patterns differed by insurance type. If certain types of physicians or certain pharmacy plans were associated with lower rates of de-adoption, the information could guide efforts to reduce the use of ineffective and unsafe treatments. Moreover, none of these studies separated the impact of the evidence on the use of different types of ESA. The decisions to prescribe EPO or DPO are likely influenced by various factors such as availability, cost, and patient preference [25, 26]. Additionally, while the TREAT trial was studied DPO, the FDA revision applied to both types of ESA, and it is plausible that the impact of the TREAT evidence differentially affected EPO and DPO prescribing.

Using administrative claims data in the US, we examined de-adoption of ESA treatment among advanced CKD stages 3–5, non-dialysis patients - those who are more likely to have anemia relative to patients in early stages of the disease [27]. First, we examined changes of EPO separately from that of DPO. Second, we examined which physician characteristics were associated with de-adoption, examining changes in prescribing in both levels and trends in response to both the trial and the FDA revision. We separately examined de-adoption in three insurance populations: commercially insured, Medicare Advantage (MA), and Medicare Fee-for-Service (FFS).

In the context of U.S. healthcare, it is crucial to consider different insurance types because of their differences in reimbursement policies and demographic characteristics. The Medicare program is the primary source of healthcare insurance for individuals aged 65 and above, while commercially insured individuals are typically younger. Within the Medicare program, enrollees may receive insurance through Medicare FFS (also known as traditional Medicare) or through Medicare Advantage. In Medicare FFS, healthcare providers bill the U.S. Centers for Medicare and Medicaid Services directly for any care that is provided. Medicare Advantage is Medicare plans offered by commercial insurance companies that contract with Medicare to provide coverage for inpatient and outpatient services as well as prescription drugs.

## Methods

### Data source

We used administrative claims data from two sources. The first source was the 2007–2015 commercial and MA administrative claims from the OptumLabs® Data Warehouse (OLDW), a comprehensive, longitudinal, real-world data asset with de-identified claims and clinical information [28]. The second source was a 20% random sample of Medicare FFS beneficiaries from 2007 to 2013. We combined the administrative data with information about physicians from Doximity®. Doximity is a data resource that allowed us to observe key physician characteristics. This database includes information from a wide range of sources, such as the National Provider Identifier Registry and state medical boards, and has been validated and used in previous literature [29–32].

### Study population

We identified three separate cohorts of patients who had at least one claim for CKD diagnosis (based on ICD 9-CM and ICD 10-CM diagnosis codes) anytime between 1/2007 and 12/2015; all were identified using medical claims in either commercial, MA or FFS data sources. We restricted the cohorts to those who had continuous medical and prescription drug coverage for the 12 months before the index diagnosis claim for CKD. In any given month, we flagged a patient to have CKD if they had at least one inpatient claim or two outpatient claims spaced more than 30 days apart with CKD diagnosis in the past 6 months. Our final analytic samples consisted of the patient-months identified to have CKD stages 3–5 without dialysis treatment. Supplemental Digital Content (Additional file 1), Section 1 provides details on the ICD 9-CM and ICD 10-CM diagnosis codes and CPT codes used to identify the study sample.

### Physician assignment

To analyze changes in ESA use by physician characteristics, we attributed each patient-month observation to the physician responsible for making decisions concerning the ESA treatment following a 2-step procedure. In the first step, we isolated medical claims for all evaluation and management (E&M) visits for each patient-month observation and divided claims into four categories based on the specialty of the associated physician: (1) nephrology, (2) internist, (3) hematology and oncology, and (4) all others. Drawing from the literature [33], we ranked these categories based on relevance to anemia treatment for patients with CKD with (1) being the most relevant and (4) being the least relevant specialty. For example, even if an internist had more E&M claims than a nephrologist in a given month, the nephrologist was attributed to the patient-month. Within each specialty category, the physician with the most E&M visits was attributed to the patient-month (ties were assigned randomly); and we carried forward that attribution, month after month, until an interruption in patient-months at risk or a change in physician with the most visits. Once each patient-month observation was attributed to a physician, we used the National Physician Identification (NPI) to merge in physician characteristics from the Doximity® database.

### Measures

The two outcome variables were dichotomous indicators for DPO and EPO use in a given patient-month observation (unit of analysis). The indicators were constructed by identifying ESA administration in outpatient claims (CPT codes “J0881” (Darbepoetin Alfa) and “J0885” (Epoetin Alfa)) or in pharmacy claims.

Patient covariates included sex, age, CKD stage, and Elixhauser comorbidity index. Physician characteristics included sex, specialty, and age (< 50, 50 or older), and physician specialty (primary care physicians (PCPs), nephrologists, and non-nephrology specialists, including internists, hematologists, and oncologists).

### Statistical analysis

We summarized DPO and EPO use, patient and physician characteristics, reporting mean (SD) or n (%) according the characteristic. Then, to assess patterns of de-adoption we estimated a series of linear segmented regression models with panel data [34–36]. First, we examined changes in ESA use among CKD patients after new evidence of unsafety and ineffectiveness from the TREAT trial publication (10/2009) and the FDA revision (6/2011). We considered three time-periods based on these events:

- Baseline period (pre-TREAT): 1/2007 (start) to 6/2009
- Period 1 (post-TREAT/pre-FDA): 2/2010 to 2/2011
- Period 2 (post-TREAT/post-FDA): 10/2011 to 12/2015 (end)

We excluded the three-month periods before and after each event to avoid capturing any anticipatory or short term effects [36]. We estimated changes in the level and the trend in use of DPO and EPO use in period 1 relative to the baseline, as well as in period 2 relative to period 1. All models adjusted for patient and physician characteristics and included calendar month dummy variables to account for seasonality. Standard errors were clustered at the patient level. Models were estimated separately for commercially insured, MA, and Medicare FFS cohorts. These models let us assess whether there were differences in de-adoption by payer; though data use agreements precluded combining these cohorts for formal testing, we report the effect magnitudes and *P*-values for each. Then, to assess whether de-adoption (changes in levels or trends) differed by physician characteristics, we estimated a second set of models which included interactions of the levels and trends with physician characteristics one at a time (for example, nephrologist or non-nephrologist). Details of the model specifications are provided in Supplemental Digital Content (Additional file 1), Section 2.

All analyses were performed with SAS, Version 9.4 (Copyright© 2002–2012 SAS Institute Inc.) and Stata 14.2 (StataCorp, College Station, TX). The study was deemed exempt from review by the University of Minnesota Institutional Review Board because the data were de-identified.

## Results

Our study included 501,287 patient-month observations for the commercially insured, 1,206,050 for MA, and 8,106,600 for Medicare FFS. Unadjusted rates of DPO use were 5.3, 3.2 and 3.5% for each insurance group respectively, while corresponding unadjusted rates of EPO use were 7.2, 5.3 and 4.5% (Table 1). For the commercially insured, the mean (SD) age was 61.7 (13.1) and the mean Elixhauser comorbidity index was 6.1 (3.0). For MA and Medicare FFS samples, the mean ages were 75.2 (8.0) and 76.3 (9.7), and the mean comorbidity index was 7.2 (3.1) and 14.9 (10.2), respectively. CKD stage 3 was the most prevalent in all three samples (75.3 to 76.0%). The majority of the observations for the commercially insured and MA patients were attributed to nephrologists (63.2 and 46.1%, respectively), while only 24.3% of the Medicare FFS observations were attributed nephrologists.

**Table 1:** Patients with Chronic Kidney Disease (CKD) stages 3–5

	Commercial	Medicare Advantage	Medicare FFS
	(*N* = 501,287)	(*N* = 1,206,050)	(*N* = 8,106,600)
***ESA use***			
EPO use (%)	7.2	5.3	4.5
DPO use (%)	5.3	3.2	3.5
***Patient characteristics***			
Female (%)	42.9	53.1	50.9
Mean Age (SD)	61.7 (13.1)	75.2 (8.0)	76.3 (9.7)
Mean Elixhauser score (SD)	6.1 (3.0)	7.2 (3.1)	14.9 (10.2)
CKD stage 3 (%)	75.3	76.0	75.8
CKD stage 4 (%)	21.5	21.8	19.9
CKD stage 5 (%)	3.2	2.2	4.3
***Physician characteristics***			
Female (%)	19.3	19.8	17.1
Completed residency under 20 years ago (%)	55.0	53.7	
Under 50 years old (%)	47.2	45.6	39.9
Nephrologist (%)	63.2	46.1	24.3
Internist (%)	11.2	22.2	27.7
Hematologist (%)	2.5	2.2	4.8
Other specialties	25.5	32.2	43.2
Number of unique patients	116,968	227,145	765,159
Number of unique physicians	46,033	56,517	302,543

**Fig. 1:**
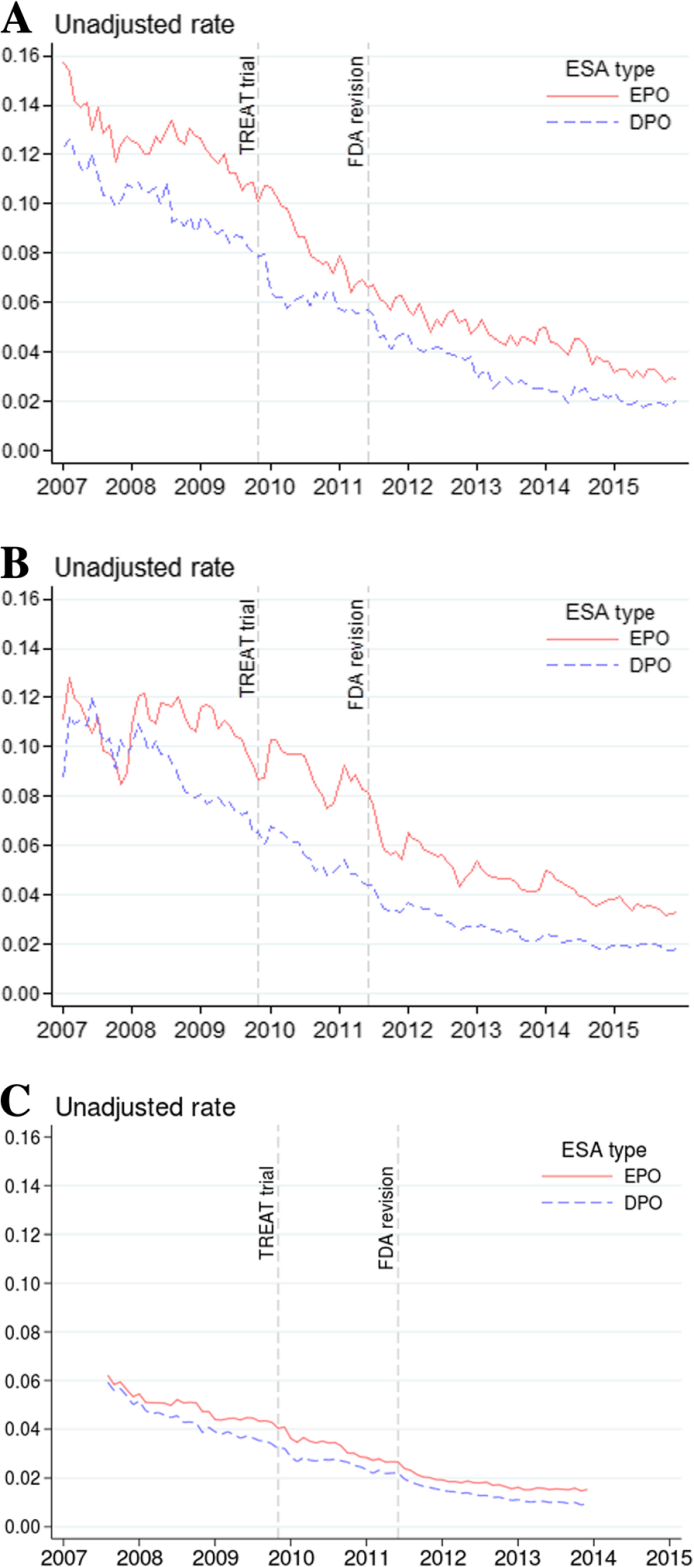
Unadjusted rates of ESA use by insurance, 2007–2015 period, CKD stages 3–5

For all three cohorts, unadjusted rates of EPO and DPO use were declining before the TREAT trial and over the entire study period (Fig. 1). For example, among the commercially insured, EPO use was 16% and DPO use was 12% in January 2007, while corresponding rates were 10 and 8% in September 2009, just before the TREAT trial.

### Changes in levels and trends of use over time by insurance cohort

#### DPO (Table 2, panel a)

Prior to the TREAT trial, DPO use declined by an average of 0.13 percentage points per month in each insurance group (all *P*-values < 0.001). Following the trial publication, commercially insured and MA did not have immediate changes in levels of use, but Medicare FFS had an immediate increase in levels of use (*P*-value < 0.01). The rate of decline among commercially insured and Medicare FFS patients had decreased (i.e, a less negative trend following trial publication), while the rate of decline for MA patients did not change. After the FDA revised its black box warning, there was an immediate decline in the levels of DPO use in all 3 groups (all *P*-values < 0.05). Commercially insured patients experienced no changes in trends, while MA and FFS patients experienced decreases in rates of decline in use (both *P*-values < 0.005).

**Table 2:** Adjusted changes in EPO and DPO rates in levels and trends following the TREAT trial publication and the FDA warning revision

	Commercial			Medicare Advantage			Medicare FFS		
	Baseline trend	Changes from		Baseline trend	Changes from		Baseline trend	Changes from	
		previous period			previous period			previous period	
		Period 1	Period 2		Period 1	Period 2		Period 1	Period 2
	(1)	(2)	(3)	(4)	(5)	(6)	(7)	(8)	(9)
**Panel A: DPO use**									
Trends	−0.13	0.10	−0.02	−0.13	0.02	0.08	−0.13	0.07	0.03
	(− 0.17, − 0.09)	(0.02, 0.17)	(− 0.09, 0.04)	(− 0.2, − 0.09)	(− 0.05, 0.09)	(0.03, 0.13)	(− 0.13, − 0.11)	(0.05, 0.08)	(0.01, 0.004)
	[< 0.001]	[0.010]	[0.478]	[< 0.001]	[0.633]	[0.003]	[< 0.001]	[< 0.001]	[< 0.001]
Levels		−0.85	−1.33		−0.245	− 0.66		0.61	−0.60
		(−1.74, 0.03)	(−2.2, −0.05)		(−1.11, 0.62)	(−1.28, −0.03)		(0.45, 0.77)	(−0.77, − 0.48)
		[0.061]	[0.003]		[0.581]	[0.040]		[< 0.001]	[< 0.001]
**Panel B: EPO use**									
Trends	−0.07	−0.18	0.19	−0.003	− 0.17	0.11	− 0.07	− 0.03	0.10
	(−0.1, − 0.03)	(− 0.27, − 0.09)	(0.11, 0.26)	(− 0.05, 0.04)	(− 0.24, − 0.09)	(0.05, 0.17)	(− 0.08, − 0.07)	(− 0.05, − 0.02)	(0.08, 0.11)
	[0.001]	[< 0.001]	[< 0.001]	[0.913]	[< 0.001]	[0.001]	[< 0.001]	[< 0.001]	[< 0.001]
Levels		−0.62	0.61		− 0.80	−0.87		0.32	−0.18
		(−1.63, 0.39)	(−0.36, 1.58)		(− 1.78, 0.18)	(− 1.65, − 0.09)		(0.15, 0.49)	(− 0.3, − 0.01)
		[0.228]	[0.215]		[0.110]	[0.028]		[< 0.001]	[0.034]
*Observations*	382,608			960,846			6,638,950		

Estimates are reported for baseline trends and changes of trends and levels of DPO and EPO use, compared to previous periods. Baseline refers to the period between Jan-2007 to June-2009; period 1 (post-TREAT/pre-FDA) is between Feb-2010 and Feb-2011; period 2 (post-TREAT/post-FDA) is between Oct-2011 and Dec-2015. All estimates were multiplied with 100 to represent percentage point changes. All samples consist of patients with CKD stage 3 to 5. All models control for patients’ sex and age and physicians’ specialty, age, sex, and experience (see text for details). Standard errors are clustered at the patient level; 95% confidence interval is reported in parentheses, and *p*-value is reported in brackets

#### EPO (Table 2, panel B)

Before the TREAT trial publication, EPO use was decreasing for commercial and Medicare FFS patients (both, *P*-values < 0.001), but was flat for MA patients. After the publication, there was no change in levels for commercially insured and MA but an increase in level for Medicare FFS (*P* < 0.001). EPO trends decreased further for all three cohorts (all *P*-values < 0.001). Following the FDA revision, MA and FFS patients experienced a decrease in levels (both *P*-values < 0.05), and all patient groups experienced an increase in trends (all *P*-values < 0.005).

### Changes in trends and levels of use over time by physician characteristics

#### DPO (Table 3)

In commercial and MA groups, we found no significant differences in changes of DPO trends between PCPs and nephrologists following the TREAT trial publication and the FDA revision. In the commercial group, decreases in level of DPO use immediately following the FDA revision were larger among nephrologists relative to PCPs (*P*-value = 0.05). In Medicare FFS, we observed that nephrologists experienced a larger immediate increase in DPO use levels (*P*-value < 0.05) after the trial and a larger increase of monthly trends after the FDA revision (*P*-value < 0.05) compared to the PCPs.

**Table 3:** Changes in DPO use levels and trends by physician characteristics

	Commercial				Medicare Advantage				Medicare FFS			
	Change from baseline to Period 1		Change from Period 1 to 2		Change from baseline to Period 1		Change from Period 1 to 2		Change from baseline to Period 1		Change from Period 1 to 2	
	Trends	Levels	Trends	Levels	Trends	Levels	Trends	Levels	Trends	Levels	Trends	Levels
	(1)	(2)	(3)	(4)	(5)	(6)	(7)	(8)	(9)	(10)	(11)	(12)
**Panel A: Nephrologists relative to PCPs (reference)**												
Difference	0.12	1.06	−0.10	−1.96	0.13	0.62	0.06	0.44	0.02	0.48	0.03	0.21
	(−0.06, 0.31)	(−1.45, 3.57)	(−0.26, 0.06)	(− 3.91, 0.00)	(0.00, 0.26)	(− 0.96, 2.21)	(− 0.05, 0.16)	(− 0.91, 1.80)	(−0.01, 0.06)	(0.02, 0.74)	(0.003, 0.06)	(−0.13, 0.56)
	[0.189]	[0.407]	[0.220]	[0.050]	[0.057]	[0.442]	[0.287]	[0.523]	[0.127]	[0.040]	[0.027]	[0.230]
**Panel B: Female physicians relative to male physicians (reference)**												
Difference	−0.23	−0.70	0.16	1.93	−0.21	− 0.62	0.14	1.65	−0.01	− 0.07	−0.01	− 0.24
	(−0.40, − 0.62)	(−2.77, 1.38)	(0.02, 0.31)	(− 0.60, 3.91)	(−0.38, − 0.03)	(− 2.79, 1.55)	(0.01, 0.27)	(0.06, 3.25)	(− 0.04, 0.02)	(− 0.44, 0.30)	(−0.03, 0.02)	(− 0.58, 0.11)
	[0.007]	[0.512]	[0.030]	[0.057]	[0.020]	[0.573]	[0.032]	[0.042]	[0.602]	[0.724]	[0.667]	[0.180]
**Panel C: Physicians under 50 years old relative to physicians over 50 years old (reference)**												
Difference	0.09	0.40	−0.03	−0.53	0.02	2.51	0.09	1.45	−0.03	0.003	0.02	0.11
	(−0.05, 0.24)	(−1.32, 2.13)	(−0.16, 0.10)	(−2.31, 1.26)	(− 0.12, 0.16)	(0.79, 4.22)	(− 0.02, 0.19)	(0.14, 2.76)	(− 0.05, − 0.002)	(−0.28, 0.29)	(0.0003, 0.04)	(−0.16, 0.38)
	[0.218]	[0.647]	[0.627]	[0.564]	[0.744]	[0.004]	[0.105]	[0.031]	[0.037]	[0.983]	[0.047]	[0.415]

Estimates are reported for changes of trends and levels of DPO use (compared to previous periods) by physician’s characteristics as well as P-value for differences across physician characteristics. Baseline refers to the period between Jan-2007 to June-2009; period 1 (post-TREAT/pre-FDA) is between Feb-2010 and Feb-2011; period 2 (post-TREAT/post-FDA) is between Oct-2011 and Dec-2015. All estimates were multiplied with 100 to represent percentage point changes. All samples consist of patients with CKD stage 3 to 5. All models control for patients’ sex and age and physicians’ specialty, age, sex, and experience (see text for details). Standard errors are clustered at the patient level; 95% confidence interval is reported in parentheses, and *P*-value is reported in brackets

In the commercial group, increases of monthly trends of DPO use were smaller for female physicians compared to male physicians after the TREAT trial (*P*-value < 0.01). DPO trends decreased after the FDA revision, but the decreases of monthly trends of DPO use were smaller for female physicians than male physicians (*P*-value < 0.05). In the MA group, increases of monthly trends of DPO use after the trial publication were also smaller among female physicians relative to male physicians (*P*-value < 0.05). Level of DPO use decreased immediately following the FDA revision, but female physicians exhibited a smaller decrease (i.e. the decrease was less negative) compared to male physicians (*P*-value < 0.05). Monthly trends of DPO use increased more for female physicians than male physicians after the FDA revision (*P*-value < 0.05). In contrast, there were no significant differences in changes of levels or trends between male and female physicians in the Medicare FFS group following the two events.

We did not observe any significant differences in changes of levels and trends between physicians under and over 50 years old following the two events in the commercial group. In contrast, we found that physicians under 50 years old in the MA group exhibited a smaller immediate decrease (i.e. the decrease was less negative) in DPO prescribing following the trial (*P*-value < 0.01) and the FDA revision (*P*-value < 0.05) compared to older physicians. In the Medicare FFS group, physicians under 50 years old experienced a larger decrease in DPO trends following the trial (*P*-value < 0.05) and a larger increase of DPO trends following the revision (*P*-value < 0.05) compared to physicians over 50 years old.

#### EPO (Table 4)

Immediate changes of EPO levels following the TREAT trial publication and the FDA revision were not significantly different between nephrologists versus PCPs in all three insurance groups. In the MA and Medicare FFS cohorts, monthly EPO trends increases following the FDA revision were larger for nephrologists (*P*-value < 0.05).

**Table 4:** Changes in EPO use levels and trends by physician characteristics

	Commercial				Medicare Advantage				Medicare FFS			
	Change from baseline to Period 1		Change from Period 1 to 2		Change from baseline to Period 1		Change from Period 1 to 2		Change from baseline to Period 1		Change from Period 1 to 2	
	Trends	Levels	Trends	Levels	Trends	Levels	Trends	Levels	Trends	Levels	Trends	Levels
	(1)	(2)	(3)	(4)	(5)	(6)	(7)	(8)	(9)	(10)	(11)	(12)
**Panel A: Nephrologists relative to PCPs (reference)**												
Difference	−0.19	1.40	0.16	0.17	−0.12	−0.58	0.15	−0.63	− 0.01	0.09	0.04	−0.13
	(−0.42, 0.03)	(−1.38, 4.18)	(−2.41, 2.75)	(−2.41, 2.75)	(−0.27, 0.04)	(−2.57, 1.40)	(0.02, 0.28)	(−2.26, 1.00)	(−0.05, 0.03)	(− 0.51, 0.33)	(0.01, 0.07)	(− 0.55, 0.29)
	[0.085]	[0.323]	[0.101]	[0.896]	[0.145]	[0.565]	[0.024]	[0.447]	[0.601]	[0.661]	[0.020]	[0.548]
**Panel B: Female physicians relative to male physicians (reference)**												
Difference	−0.08	−0.80	0.09	0.50	0.24	2.11	−0.06	0.03	−0.02	−0.19	0.03	0.05
	(−0.29, 0.14)	(−3.38, 1.78)	(−0.09, 0.28)	(−1.96, 2.96)	(0.04, 0.44)	(−0.31, 4.54)	(− 0.21, 0.09)	(− 1.93, 1.99)	(− 0.06, 0.02)	(−0.60, 0.23)	(− 0.004, 0.06)	(− 0.35, 0.44)
	[0.480]	[0.543]	[0.324]	[0.691]	[0.017]	[0.088]	[0.432]	[0.974]	[0.326]	[0.373]	[0.091]	[0.810]
**Panel C: Physicians under 50 years old relative to physicians over 50 years old (reference)**												
Difference	0.00	0.75	0.05	0.34	0.06	−2.04	−0.11	−0.81	−0.02	0.08	0.03	0.19
	(−0.17, 0.17)	(−1.20, 2.70)	(−0.10, 0.20)	(−1.58, 2.25)	(− 0.09, 0.21)	(−3.95, − 0.12)	(−0.23, 0.01)	(− 2.38, 0.75)	(− 0.04, 0.01)	(−0.23, 0.40)	(0.003, 0.05)	(−0.11, 0.49)
	[0.967]	[0.451]	[0.517]	[0.732]	[0.422]	[0.037]	[0.081]	[0.309]	[0.231]	[0.614]	[0.025]	[0.211]

Estimates are reported for changes of trends and levels of EPO use (compared to previous periods) by physician’s characteristics as well as P-value for differences across physician characteristics. Baseline refers to the period between Jan-2007 to June-2009; period 1 (post-TREAT/pre-FDA) is between Feb-2010 and Feb-2011; period 2 (post-TREAT/post-FDA) is between Oct-2011 and Dec-2015. All estimates were multiplied with 100 to represent percentage point changes. All samples consist of patients with CKD stage 3 to 5. All models control for patients’ sex and age and physicians’ specialty, age, sex, and experience (see text for details). Standard errors are clustered at the patient level; 95% confidence interval is reported in parentheses, and *P*-value is reported in brackets

Similarly, there were no significant differences between male and female physicians in immediate changes of EPO levels following the TREAT trial and the FDA revision in all three insurance groups. Changes of monthly trends after the two events were not significantly different between the female and male physicians except for the MA group, where EPO trends decreased after the TREAT trial, but the decrease was smaller (i.e. less negative) for female physicians relative to male physicians (*P*-value < 0.05).

Immediate changes in EPO levels and monthly trends following the trial and the FDA revision were also not significantly different between physicians under and over 50 years old in the commercial group. In the MA group, physicians under 50 years old exhibited a larger immediate decrease in EPO prescribing following the trial publication (*P*-value < 0.05). In the Medicare FFS group, physicians under 50 years old also had a larger increase in monthly EPO trends (*P*-value < 0.05) after the FDA revision.

## Discussion

In this examination of ESA use in three insurance cohorts, we found that DPO and EPO use were both already declining prior to the TREAT trial publication, and they continued to decrease following the TREAT trial and the FDA black box warning revision. While this was consistent with prior research, we also found differences in how the trends and levels changed: by treatment, by insurance group, and by physician characteristics. Consistent with expectations, the decline in EPO use became steeper after the TREAT trial across all three insurance groups, but surprisingly, the decline in DPO use slowed (the trend was less negative) after the TREAT trial in commercially insured and Medicare FFS groups and did not change in the MA group. One critique of the trial is that more patients from the placebo group received intravenous iron than patients assigned to darbepoetin alfa due to a lack of an iron administration protocol; this limitation might have led to skepticism from physicians and a lack of response to the evidence of the trial [15]. Moreover, after the FDA revision, the decline in DPO use slowed again in the MA and Medicare FFS, though not in the commercial group, while the decline in EPO use slowed in all three groups.

Notably, although the decline in DPO prescribing slowed after both the TREAT trial and the FDA revision, DPO use dropped immediately after the FDA revision in all three insurance groups; this suggests both that the subsequent weaker decline (relative to the trend prior to the FDA revision) may reflect in part lower overall use, and also that the FDA revision was viewed as stronger evidence relative to the TREAT trial publication associated with a decrease in use. We observed the similar immediate decline for EPO use after the FDA revision as well.

Differences in de-adoption across insurance cohorts were minor and with no consistent relationship between insurance group and changes in levels or trends. That we found fewer changes in levels and trends in the commercial cohort may reflect the smaller sample size, especially compared with the FFS group (with 34 times as many patients), but even that cohort had more than half a million patients. More likely these differences reflect differences in providers who treat more or fewer commercial, FFS or MA patients.

With regards to providers, we found that both the slower decline trend in DPO use as well as the faster decline trend in EPO use after the TREAT trial were generally driven by nephrologists to a greater extent. We also observed some differences in responses to the trial and the warning revision by physician’s gender and age, but the differences were not consistent for DPO and EPO and for different insurance populations. These results suggest that physician specialty have a dominant role in prescribing decisions, and that specializations with higher use of treatments were more responsive to new evidence of unsafety and ineffectiveness. This may be because the patient populations attributed to specialists for a given condition, in this case nephrologists, were more severe in unobserved ways.

Our findings are broadly consistent with prior research on de-adoption of treatments, which has found variation across providers in how rapidly treatments fall out of use in the wake of new evidence. Borne et al. [37] found that variation across institutions increased with increasing de-adoption of defibrillation following evidence of risk, indicating that providers de-adopted at different rates; they attribute this to different institutional practices. Bekelis et al. [38] found that more experienced physicians reduced their use of carotid revascularization more quickly than other physicians, while higher volume physicians reduced their use more slowly. More generally, van Dulmen et al. [39] identified 263 barriers to de-adoption of treatments, of which the majority were physician factors. Thus, there are large variety of factors that may systematically influence the de-adoption of EPO and DPO, which suggests the need for systematic efforts to promote de-adoption [40].

There are several limitations to this study. First, our patient populations were identified using diagnosis codes and medication use in administrative claims. It also only included patients with non-dialysis CKD stages 3 to 5, so our results may not be generalized to other types of patients. Moreover, our analyses could not account for many unobserved factors of ESA use such as treatment cost, patient preference, and ESA treatment appropriateness. The FDA warning indicated that ESA treatment can be considered if the hemoglobin level was less than 10 g/dL, the rate of hemoglobin declined, and reducing the risk of alloimmunization and/or red blood cell transfusion was the goal. These unobserved conditions would influence physician decisions whether to prescribe ESA treatment for patients.

Our data did not cover the period before 2007 so our analyses could not account for earlier studies on the unsafety of ESA treatment, including the NHCT, CHOIR, and CREATE trials, although other studies found that ESA prescribing trends substantially decreased in 2007 as a response to these events [18, 19]. However, we note that our segmented regression approach controls for monthly trends and levels of ESA use between January 2007 and the TREAT trial publication in 2009. Although there are other events that also influenced ESA prescribing decisions such as the Medicare reimbursement reform in 2011 and the peritoneal dialysis solution shortage in 2014 [22, 23, 41], they mainly concerned patients on dialysis, who were not part of our study populations; therefore, we do not expect our results to be affected by these events. Lastly, it is possible that physicians also changed ESA dosage in response to the FDA warning; however, we did not assess changes in ESA dosage because our analysis was conducted at the patient-month level, while an adequate dosing is usually measured over consecutive days or weeks of therapy for a patient, depending on the half-life of the drug.

In summary, we found that DPO and EPO prescribing started decreasing in 2007 and continued to decline over the study period. The impact of the TREAT trial and of the FDA revision was limited and inconsistent, with a de-adoption rates of decline slowing for DPO for some payers. These findings have implications for the treatment of CKD both inside and outside of the US, as ESA continues to be the most common therapy to treat anemia among CKD patients [7].

Our study is also relevant to a growing literature on physicians’ decisions to de-adopt treatments in light of new clinical evidence suggesting that a previously-approved treatment is ineffective or unsafe [37–40, 42–44]. While existing studies mostly focus on measuring the rate of de-adoption or reductions in prescribing, there is less evidence regarding variation in de-adoption [38, 44]. Our findings indicate that de-adoption of unsafe treatments varies across insurance types and across physician specialties and characteristics. It is important to take these variations into account when designing practice guidance to promote efficient de-adoption.

## Supplementary Information

**Additional file 1.**

## Data Availability

We are using proprietary data accessed from OptumLabs approved a Detailed Research Application (DRA) and administrative Medicare files from CMS that are approved under a Data Use Agreement (DUA) purchased from CMS for this project. Ass such we are not authorized to share any data, but we are happy to suggest researchers interested the appropriate steps to acquire data on their own for their specific DUA.

## Abbreviations

ESA: Erythropoiesis-stimulating agents
CKD: Chronic kidney Disease
EPO: Epoetin alfa
DPO: Darbepoetin alfa
TREAT: Trial to Reduce Cardiovascular Events with Aranesp Therapy
FDA: Food and Drug Administration
MA: Medicare Advantage
FFS: Fee-for-Service
OLDW: OptumLabs® Data Warehouse
E&M: Evaluation and management
NPI: National Physician Identification
PCP: Primary care physicians
SD: Standard deviation
