# Supplementary material for: Uptake of evidence by physicians: De-adoption of erythropoiesis-stimulating agents after the TREAT trial
Source: BMC Nephrol. 2021 Aug 21;22:284. doi: 10.1186/s12882-021-02491-y (PMC8379779; doi:10.1186/s12882-021-02491-y)
Supplement: Supplementary file 1 — Additional file 1. [file 12882_2021_2491_MOESM1_ESM.docx]

Supplemental Digital Content.

Uptake of evidence by physicians: De-adoption of erythropoiesis-stimulating agents after TREAT trial showed they are ineffective and unsafe.

Table of Contents.

Section 1: ICD 9-CM and ICD 10-CM diagnosis codes to identify chronic kidney disease, and CPT codes to identify dialysis treatment.

Section 2: Segmented regression statistical model specifications.

Section 1: ICD 9-CM and ICD 10-CM diagnosis codes to identify chronic kidney disease, and CPT codes to identify dialysis treatment.

| **1. Identify chronic kidney disease** | | |
| --- | --- | --- |
| **ICD 9-CM:** 016.0, 095.4, 189.0, 189.9, 223.0, 236.91, 250.4, 271.4, 274.1, 283.11, 403, 404, 440.1, 442.1, 572.4, 581, 582, 583, 584, 585, 586, 585.9, 285.21 |  | **ICD 10-CM:** A18.1, A52.75, C64.9, C68.9, D30.00, D41.00, D41.20, E11.2, E74.8, M10.3, D59.3, I12, I13, I70.1, I72.2, K76.7, N04, N03, N05, N17, N19, N26.9, N25, N13.30, O10.4, O26.83, Q61.3, Q61.2, Q61.19, Q61.4, Q61.5, Q61.02, Q61.8, Q62.3, R94.4, N18.1, N18.2, N18.3, N18.4, N18.5, 18.6, N18.9, D63.1 |
| **2. Identify chronic kidney disease stages 3, 4 and 5** | | |
| **ICD 9-CM:** 585.3, 585.4, 585.5 |  | **ICD 10-CM:** N18.3, N18.4, N18.5 |
| **3. Identify dialysis treatment**  **CPT codes:** 90935, 90,937, 90,945, 90,947, 90,989, 90,993, 90,999. | | |

**Section 2: Segmented regression statistical model specifications**

We examined changes in EPO and DPO use among chronic kidney disease patients after new evidence of unsafety and ineffectiveness from the TREAT trial publication (October 2009) and the FDA revision of label warning for EPO and DPO (June 2011), using segmented regression approach. We considered three time-periods based on these events:

- Baseline period (pre-TREAT): 1/2007 (start) to 6/2009
- Period 1 (post-TREAT/pre-FDA): 2/2010 to 2/2011
- Period 2 (post-TREAT/post-FDA): 10/2011 to 12/2015 (end)

Let $\phi$ denotes these two events and $Y_{ijm}$ denotes ESA use outcome of patient $i$ of physician $j$ in month-year $m$, we estimated the following linear regression model:

$Y_{ijm}=\sum_{\phi=\{TREAT;FDA\}} \left[ \alpha^{\phi}{Post}_{m}^{\phi}+\beta^{\phi}\left( {Post}_{m}^{\phi}\times t_{m}^{\phi} \right) \right] +\gamma m+\boldsymbol{Z}_{\boldsymbol{ijm}}^{\boldsymbol{'}}\boldsymbol{\theta}+\epsilon_{ijm}$ (1)

where $Post_{m}^{\phi}$ were binary variables indicating whether month-year $m$ was after event $\phi$ and $t_{m}^{\phi}$ measures the number of months since event $\phi$, $m$ is the typical linear trend variable, $\boldsymbol{Z}_{\boldsymbol{ijm}}^{\boldsymbol{'}}$ was a vector of patient and physician characteristics as controls, and $\epsilon_{ijm}$ was the error term. In this model, $\alpha^{TREAT}$ and $\beta^{TREAT}$ represent changes of drug use in levels and in trends, respectively, following the TREAT trial publication relative to the baseline period; $\alpha^{FDA}$ and $\beta^{FDA}$ represent changes of drug use in levels and in trends, respectively, following the FDA warning revision relative to period 1

We extended Eq. (1) to examine whether the changes in EPO and DPO use varied by each physician characteristics by estimating the linear regression of:

$Y_{ijm}=\sum_{\phi=\{TREAT;FDA\}} \left[ \alpha^{\phi}{Post}_{m}^{\phi}+\beta^{\phi}\left( {Post}_{m}^{\phi}\times t_{m}^{\phi} \right)+\delta^{\phi}\left( {Post}_{m}^{\phi}\times t_{m}^{\phi}\times C_{ij} \right)+\psi^{\phi}\left( C_{ij}\times Post_{m}^{\phi} \right) \right] +\gamma m+\sigma C_{ij}+\tilde{\boldsymbol{C}_{\boldsymbol{ij}}^{\boldsymbol{'}}}\boldsymbol{\lambda+}\boldsymbol{X}_{\boldsymbol{im}}^{\boldsymbol{'}}\boldsymbol{\theta}+\epsilon_{ijm}$ (2)

where $C_{ij}$ was a binary variable indicating the physician characteristics of interest, e.g. nephrologist: yes (1) or no (0), $\tilde{\boldsymbol{C}_{\boldsymbol{ij}}^{\boldsymbol{'}}}$ denotes a vector of other physician characteristics and $\boldsymbol{X}_{\boldsymbol{im}}^{\boldsymbol{'}}$ denotes a vector of patient characteristics as controls. For physicians with characteristics $C_{ij}=0$, $\alpha^{\phi}$ and $\beta^{\phi}$ measure changes of treatment use following event $\phi$ relative to the previous period. For physicians with characteristics $C_{ij}=1$, changes in levels following event $\phi$ are measured by $(\alpha^{\phi}+\psi^{\phi})$ and changes in trends are measured by $(\beta^{\phi}+\sigma^{\phi})$. All models adjusted for binary indicators for each month to account for seasonality. Standard errors were clustered at the patient levels in all models
